# Gilteritinib, a FLT3/AXL inhibitor, shows antileukemic activity in mouse models of FLT3 mutated acute myeloid leukemia

**DOI:** 10.1007/s10637-017-0470-z

**Published:** 2017-05-17

**Authors:** Masamichi Mori, Naoki Kaneko, Yoko Ueno, Masaki Yamada, Ruriko Tanaka, Rika Saito, Itsuro Shimada, Kenichi Mori, Sadao Kuromitsu

**Affiliations:** 1 Research Program Management Office, Drug Discovery Research, Astellas Pharma Inc., 21 Miyukigaoka, Tsukuba-shi, Ibaraki, 305-8585 Japan; 2 Pharmacology Research Division, Astellas Research Technologies Co., Ltd, 21 Miyukigaoka, Tsukuba-shi, Ibaraki, Japan

**Keywords:** Acute myeloid leukemia, Fms-like tyrosine kinase 3, Mutation, Protein kinase inhibitors, Xenograft antitumor assays

## Abstract

Advances in the understanding of the molecular basis for acute myeloid leukemia (AML) have generated new potential targets for treatment. Fms-like tyrosine kinase 3 (FLT3) is one of the most frequently mutated genes in AML and mutations in this gene are associated with poor overall survival. AXL plays a role in the activation of FLT3 and has been implicated in the pathogenesis of AML. The studies reported here evaluated the ability of a novel FLT3/AXL inhibitor, gilteritinib, to block mutated FLT3 in cellular and animal models of AML. Initial kinase studies showed that gilteritinib, a type I tyrosine kinase inhibitor, was highly selective for both FLT3 and AXL while having weak activity against c-KIT. Gilteritinib demonstrated potent inhibitory activity against the internal tandem duplication (FLT3-ITD) and FLT3-D835Y point mutations in cellular assays using MV4–11 and MOLM-13 cells as well as Ba/F3 cells expressing mutated FLT3. Gilteritinib also inhibited FLT3-F691 mutations, although to a lesser degree, in these assays. Furthermore, gilteritinib decreased the phosphorylation levels of FLT3 and its downstream targets in both cellular and animal models. In vivo, gilteritinib was distributed at high levels in xenografted tumors after oral administration. The decreased FLT3 activity and high intratumor distribution of gilteritinib translated to tumor regression and improved survival in xenograft and intra-bone marrow transplantation models of FLT3-driven AML. No overt toxicity was seen in mouse models treated with gilteritinib. These results indicate that gilteritinib may be an important next-generation FLT3 inhibitor for use in the treatment of FLT3 mutation-positive AML.

**Electronic supplementary material:**

The online version of this article (doi:10.1007/s10637-017-0470-z) contains supplementary material, which is available to authorized users.

## Introduction

Acute myeloid leukemia (AML) is a rapidly progressing hematologic cancer characterized by loss of normal differentiation and uncontrolled proliferation of hematopoietic progenitor cells in the bone marrow [1]. This dysregulation of blood cell production leads to anemia, neutropenia, and thrombocytopenia [1]. First-line treatment for patients (aged ≤60 years) with AML often begins with a cytarabine- and anthracycline-based chemotherapy regimen through remission induction and post-remission phases. While the complete remission rate for patients who undergo this line of treatment is over 70% [1], the 5-year survival rate is still only 50% [2]. Age and leukemia cytogenetics affect patient response rate [2–5], and therapeutic outcomes are worse for patients who are not eligible for the intensive treatment or who are relapsed/refractory to front-line therapy [6, 7]. A number of advances have been made in understanding the genetics behind AML [2, 3] that may lead to new opportunities for the development of targeted therapies.

Fms-like tyrosine kinase 3 (FLT3) is a member of the class III receptor tyrosine kinase (TK) family that is normally expressed on the surface of hematopoietic progenitor cells [8]. The FLT3 receptor plays an important role in proliferation, survival, and differentiation of multipotent stem cells [8], and mutations of FLT3 are the most common molecular alteration in AML. Internal tandem duplications (ITDs) at the juxtamembrane domain within FLT3 are present in 25–30% of newly diagnosed AML cases [9–12], representing the most frequent FLT3-activating mutation. Activating point mutations within the FLT3 TK domain (TKD) are also observed in patients with AML, but with less frequency than ITD mutations [11, 13]. These activating mutations are oncogenic and are associated with poor prognosis, higher relapse rate, more rapid time to relapse, reduced disease-free survival, and reduced overall survival, although the prognostic impact for FLT3-TKD mutations is controversial [11, 12, 14, 15]. Overall, these findings suggest that FLT3 is a potential therapeutic target for patients with AML who are harboring a FLT3 mutation.

Similar to FLT3, AXL (a member of the TAM family of receptor TKs) has transforming properties and has been identified as a potential therapeutic target for AML [16, 17]. Preclinical studies showed that inhibition of AXL blocks proliferation of FLT3 mutant and FLT3 wild-type AML cells and also suppresses the leukemic burden of FLT3-ITD^+^ AML in both a subcutaneous xenograft model and a leukemia engraftment model [16–18]. Furthermore, activated AXL may be required for resistance to FLT3 inhibitors [19]. Therefore, a new chemical entity that targets both FLT3 and AXL may provide a novel treatment option for AML.

Gilteritinib is a small-molecule FLT3/AXL inhibitor with a structure based on a pyrazine carboxamide scaffold. The research described here aimed at characterizing the activity of gilteritinib against FLT3-driven AML in preclinical models. The inhibitory effects of gilteritinib against FLT3 were assessed in cell-free systems and cellular assays, and its antitumor activity and survival benefits were evaluated in FLT3-driven tumors in mice.

## Materials and methods

### Compounds, cell lines, and antibodies

Small molecule TK inhibitors (TKI) gilteritinib hemifumarate (gilteritinib; ASP2215) and quizartinib were synthesized by Astellas Pharma Inc. (Tokyo, Japan). Both gilteritinib and quizartinib were dissolved in DMSO or suspended in 0.5% methylcellulose for in vitro or in vivo experiments. MV4–11 cells were purchased from the American Type Culture Collection (ATCC, Manassas, VA); MOLM-13 cells were purchased from the German Collection of Microorganisms and Cell Cultures (Braunschweig, Germany). MV4–11-luc cells, which exogenously express firefly luciferase, were prepared at Astellas Pharma Inc. (a detailed protocol for the generation of the constructs is found in the online resource). MV4–11-AXL cells, which exogenously express AXL, were also prepared at Astellas Pharma Inc. Ba/F3 cells expressing FLT3-ITD, FLT3-D835Y, FLT3-ITD-D835Y, FLT3-ITD-F691 L, or FLT3-ITD-F691I mutations (FLT3-ITD_Ba/F3, FLT3-D835Y_Ba/F3, FLT3-ITD-D835Y_Ba/F3, FLT3-ITD-F691L_Ba/F3, FLT3-ITD-F691I_Ba/F3) were established by Astellas Pharma Inc. The following antibodies were used for immunoblotting: FLT3 (Abcam, Cambridge, MA); phosphorylated pan-tyrosine (Merck Millipore, Billerica, MA); p44/42 MAPK (ERK1/2), phospho-p44/42 MAPK (ERK1/2) (Thr202/Tyr204) (197G2) (E10), AKT, phospho-AKT (Ser473) (193H12), and AXL (C44G1) (Cell Signaling Technology, Danvers, MA); STAT5 and phospho-STAT5 (Y694) (BD Biosciences, Franklin Lakes, NJ); phospho-AXL (Y779) (MAB6965, R&D Systems, Minneapolis, MN); and β-actin (Sigma-Aldrich, St. Louis, MO).

### Kinase inhibitory assays

The kinase inhibitory activity of gilteritinib was tested against a panel of 78 TKs (Table S1) using ATP concentrations that were approximately equal to the K_m_ value for each kinase in a TK-ELISA or off-chip mobility shift assay (MSA) at Carna Biosciences, Inc. (Kobe, Japan). These assays were conducted according to the manufacturer’s instructions. Initially, two concentrations of gilteritinib (1 nM and 5 nM) were tested to assess each compound’s inhibitory effect on TK activity. Further studies were then conducted using a dose range of gilteritinib to determine IC_50_ values for kinases in which activity was inhibited by >50% with 1 nM gilteritinib as well as for c-KIT. TK-ELISA and MSA assays were used to conduct IC_50_ studies for FLT3, LTK, AXL, and c-KIT; the HTRF® KinEASE™-TK assay (Sceti Medical Labo, Tokyo, Japan) was performed according to the manufacturer’s protocol to assess the IC_50_ value of echinoderm microtubule-associated protein-like 4-ALK (EML4-ALK; three individual experiments for each kinase).

### Cell viability

The effect of gilteritinib on MV4–11 and MOLM-13 cells was assessed using the CellTiter-Glo® Luminescent Cell Viability Assay (Promega, Madison, WI). Subsequent studies were conducted to examine the effect of gilteritinib and quizartinib on Ba/F3 cells expressing either FLT3-ITD, FLT3-D835Y, FLT3-ITD-D835Y, FLT3-ITD-F691 L, or FLT3-ITD-F691I. A detailed protocol is provided in the online resource.

### Immunoprecipitation and immunoblotting

FLT3 immunoprecipitation and immunoblotting for all targets were performed following standard protocols. Detailed methods are provided in the online resource.

### Animal models for in vivo studies

All animal experimental procedures were approved by the Institutional Animal Care and Use Committee of Astellas Pharma Inc., and the Tsukuba Research Center of Astellas Pharma Inc. was awarded accreditation status by AAALAC International. Mice were maintained on standard diet and water throughout experimental procedures.

### In vivo mouse xenograft model

Details on the cell engraftment for the mouse xenograft model and the dosing protocols are provided in the online resource.

### Pharmacokinetics

Nude mice subcutaneously xenografted with MV4–11 cells received gilteritinib suspended in a 0.5% methylcellulose solution as an oral single dose. Blood samples were collected at protocol-specified time points from the inferior vena cava using a syringe with EDTA-2Na, and plasma samples were prepared by centrifugation (*n* = 3 per group; five plasma samples showed abnormal results and were not used for data analysis). Tumor samples were also collected from each mouse and tumor weight was measured. The plasma and tumor concentrations of gilteritinib were measured using high-performance liquid chromatography-tandem mass spectrometry at Nemoto Science (Ibaraki, Japan). Standard pharmacokinetic parameters (C_max_, T_max_, and AUC_t_) were calculated from the mean concentrations of gilteritinib using WinNonlin V6.1 (Certara, Princeton, NJ).

### In vivo xenograft mouse studies for gilteritinib kinase inhibitory activity

The in vivo gilteritinib kinase inhibitory activity was assessed using tumor protein lysates from the xenograft mouse model in an ELISA for phosphorylated-FLT3 and total FLT3. Gilteritinib inhibitory activity was also confirmed by analyzing phosphorylated-STAT5 levels. Detailed protocols can be found in the online resource.

### In vivo xenograft studies for gilteritinib antitumor activity

Once tumor growth had been confirmed, mice xenografted with MV4–11 cells were monitored for 28 days; mice xenografted with Ba/F3 cells expressing FLT3 mutations were monitored for 7 days. Tumor diameter was measured using a caliper, and tumor volume was determined by calculating the volume of an ellipsoid using the formula: length × width^2^ × 0.5. Body weight was measured using a standard balance. Data are expressed as mean ± SEM from *n* = 6 mice/group (MV4–11) and *n* = 5 mice/group (Ba/F3).

### Intra-bone marrow transplantation model

In the intra-bone marrow transplantation (IBMT) model, MV4–11-luc cells (1 × 10^6^ cells/mouse) were injected into the bone marrow of the left tibia of female NOD-SCID mice (day 0) following the protocol detailed by Lee et al. [20]. Tumor growth was monitored by bioluminescent imaging of the whole body with an IVIS Spectrum (PerkinElmer, Waltham, MA). After confirming tumor cell engraftment at day 14, mice were orally administered with once-daily vehicle control or gilteritinib at 30 mg/kg from day 15 to day 70 (*n* = 10). Tumor growth was monitored once a week during the dosing period and then every other week until day 100. Survival was also monitored daily until day 168.

### Computational modeling

Docking simulation of gilteritinib with FLT3 was performed using the docking software GLIDE implemented in Maestro version 9.7 (Schrodinger, LLC, New York, NY). The coordinate of ligand–protein binding for FLT3 was modeled based on the ATP-binding form of cKIT, which had the most similar kinase domain amino acid sequence in the Protein Data Bank (PDB) (PDB ID: 1PKG, chain A) and, therefore, was used as a surrogate for FLT3. The homology modeling was done by the modeling software MOE (Chemical Computing Group Inc., Montreal, Quebec, Canada). Hydrogen atoms were added using the computer program Protonate3D implemented in MOE. The docking mode with the highest docking score was employed. All molecular visualization was produced by MOE.

### Statistical analyses

For the in vivo subcutaneous xenograft mouse model, values are expressed as mean ± SEM. Tumor volume and body weight on day 28 in the gilteritinib-treated groups were compared with those in the control group using Dunnett’s test. For the IBMT model, the bioluminescence value on day 42 from the gilteritinib-treated group was compared with that from the control group using Student’s t-test. The median survival times were compared using the log-rank test. *P* < 0.05 was considered significant. SAS software (SAS Institute Inc., Cary, NC), Microsoft Excel (Microsoft, Redmond, WA), and GraphPad Prism (GraphPad Software, La Jolla, CA) were used for data processing.

## Results

### Gilteritinib inhibitory activity

**Fig. 1:**
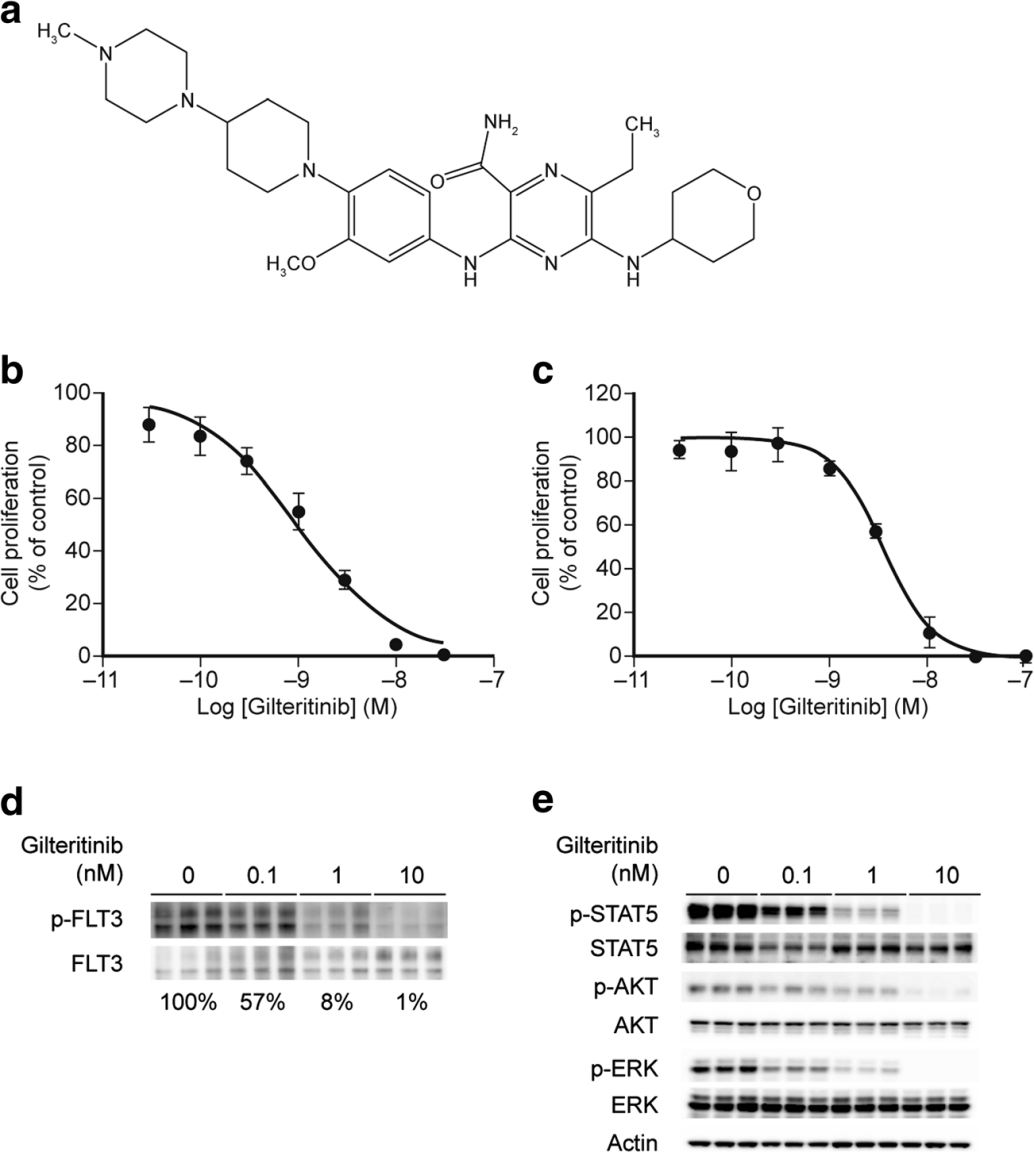
Gilteritinib inhibits cell growth in AML cells and blocks phosphorylation of FLT3 and its downstream targets. **a** Structure of gilteritinib (ASP2215). **b** MV4–11 cells were treated with DMSO or increasing concentrations of gilteritinib for 5 days, and cell viability was measured using CellTiter-Glo. A representative result from three independent experiments is shown. Data are presented as mean ± SEM (quadruplicate). **c** MOLM-13 cells were treated in the same manner as MV4–11. A representative result from three independent experiments is shown. Data are presented as mean ± SEM (quadruplicate). **d** Immunoprecipitation and immunoblot for phosphorylated FLT3 and total FLT3 in MV4–11 cells treated with DMSO or increasing concentrations of gilteritinib for 2 h. Blots from one study, which was done in triplicate, are shown. Densitometry values are listed below each treatment group. **e** Immunoblot for phosphorylated AKT, ERK, and STAT5 in gilteritinib-treated MV4–11 cells. Blots from one study, which was done in triplicate, are shown

Gilteritinib (structure provided in Fig. 1a) inhibited the activity of eight of the 78 tested kinases by over 50% at concentrations of either 1 nM (FLT3, LTK, ALK, and AXL) or 5 nM (TRKA, ROS, RET, and MER) (Table S1). The IC_50_ values were 0.29 nM for FLT3 and 0.73 nM for AXL. Gilteritinib inhibited FLT3 at an IC_50_ value that was approximately 800-fold more potent than the concentration required to inhibit c-KIT (230 nM; Table 1).

**Table 1:** Inhibitory activity of gilteritinib against various tyrosine kinases

Kinase	IC_50_ (nM) (95% CI)
FLT3	0.29 (0.26–0.32)
LTK	0.35 (0.29–0.43)
AXL	0.73 (0.51–1.0)
EML4-ALK	1.2 (0.68–2.0)
c-KIT	230 (190–280)

Inhibitory assays for tyrosine kinases were conducted using TK-ELISA, off-chip MSA, or HTRF® KinEASE™-TK. Geometric mean IC_50_ values were determined from 3 experiments

We next evaluated the antiproliferative activity of gilteritinib against MV4–11 and MOLM-13 cells, which endogenously express FLT3-ITD. After 5 days of treatment, gilteritinib inhibited the growth of MV4–11 and MOLM-13 cells with mean IC_50_ values of 0.92 nM (95% CI: 0.23–3.6 nM) and 2.9 nM (95% CI: 1.4–5.8 nM), respectively (Fig. 1b, c). Growth suppression of MV4–11 cells was accompanied by inhibition of FLT3 phosphorylation. Relative to vehicle control cells, phosphorylated FLT3 levels were 57%, 8%, and 1% after 2 h of treatment with 0.1 nM, 1 nM, and 10 nM gilteritinib, respectively (Fig. 1d). In addition, doses as low as 0.1 nM or 1 nM resulted in the suppression of phosphorylated ERK, STAT5, and AKT, all of which are downstream targets of FLT3 activation (Fig. 1e, Table S2). To investigate the effects of gilteritinib on AXL inhibition, MV4–11 cells that expressed exogenous AXL were treated with gilteritinib. At concentrations of 1 nM, 10 nM, and 100 nM for 4 h, gilteritinib treatment decreased phosphorylated AXL levels by 38%, 29%, and 22%, respectively (Fig. S1).

### Pharmacokinetic profile and pharmacodynamic effects of gilteritinib in an MV4–11 xenograft mouse model

**Fig. 2:**
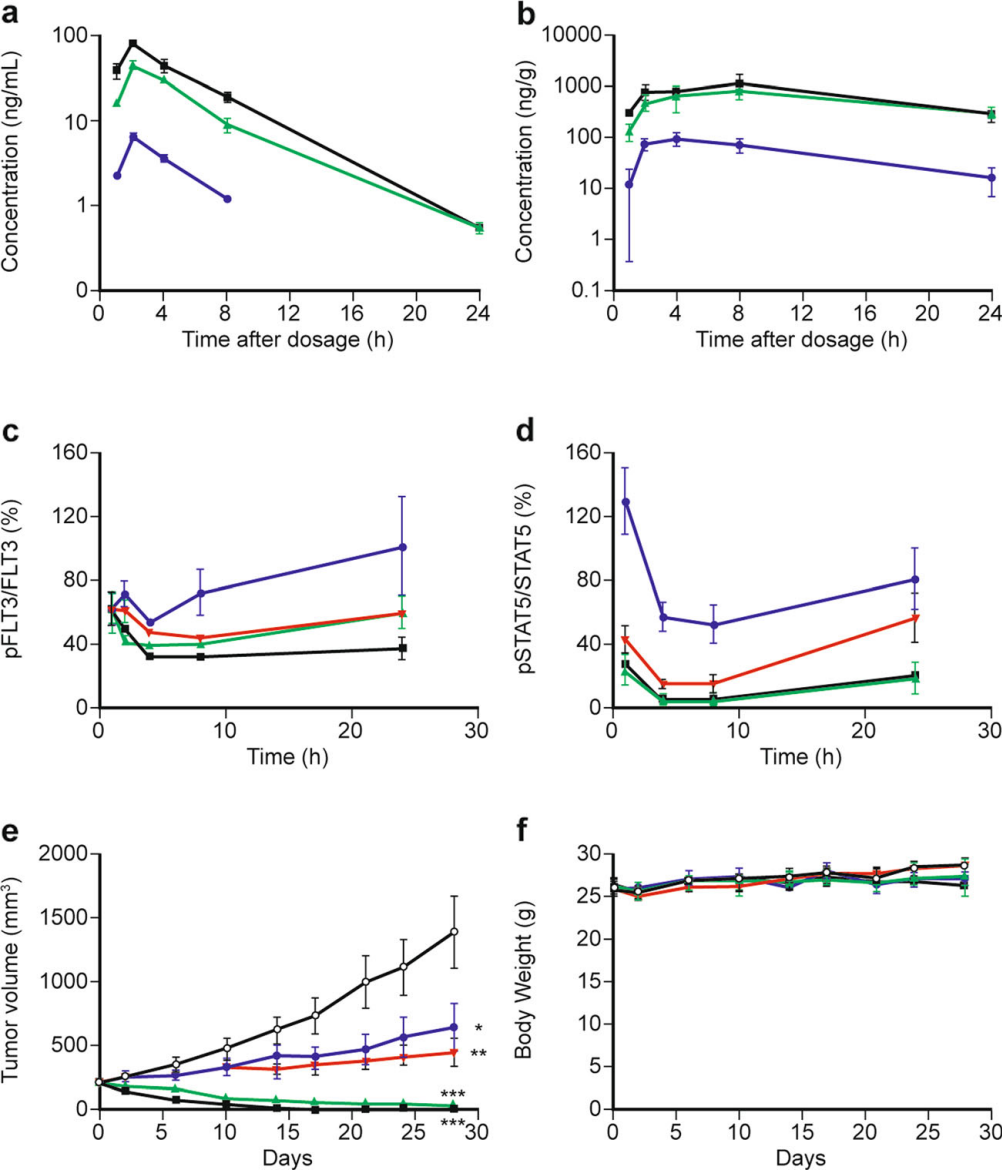
Antitumor activity of gilteritinib in an MV4–11 xenograft AML mouse model. **a** Plasma concentrations of gilteritinib were determined by high-performance liquid chromatography-tandem mass spectrometry from nude mice subcutaneously xenografted with MV4–11 cells and treated with a single oral dose of gilteritinib. Data are presented as mean ± SD for two or three animals. **b** Tumor concentrations of gilteritinib were determined by high-performance liquid chromatography-tandem mass spectrometry from nude mice subcutaneously xenografted with MV4–11 cells and treated with a single dose of gilteritinib. Data are presented as mean ± SD for three animals. Symbols represent treatment groups: 1 mg/kg (), 6 mg/kg (), 10 mg/kg (■). **c** and **d** Male mice xenografted with MV4–11 cells were orally treated with either vehicle control or increasing concentrations of gilteritinib. Protein lysates were collected over a 24 h time course for assessment of (**c**) phospho-FLT3 to total FLT3 as determined by ELISA or (**d**) phospho-STAT5 and total STAT5 as determined by immunoblot. Data are presented as the ratio of phosphorylated protein levels normalized to total protein levels relative to vehicle control-treated lysates. **e** and **f** Male mice xenografted with MV4–11 cells were orally treated with either vehicle control or increasing concentrations of gilteritinib over a 28-day period to examine the effect of drug on (**e**) tumor volume and (**f**) body weight. Data presented as mean ± SEM for *n* = 6 mice/group. Symbols represent treatment groups: 1 mg/kg (), 3 mg/kg (), 6 mg/kg (), 10 mg/kg (■). *P < 0.05, **P < 0.01, and ***P < 0.001 compared with the value of the control group on Day 28 (Dunnett’s test)

The maximal plasma concentrations of gilteritinib were observed 2 h after a single oral administration of gilteritinib at 1 mg/kg, 6 mg/kg, and 10 mg/kg to MV4–11 xenografted mice. By contrast, the maximal intratumor concentrations were observed 4 h (1 mg/kg) or 8 h (6 mg/kg and 10 mg/kg) after dosing. C_max_ and AUC_t_ in plasma and tumors increased with increasing doses between 1 mg/kg and 10 mg/kg. The concentration in tumors was higher than that in plasma at each time point (Fig. 2a–b, Table S3). Gilteritinib dose ranges for cell viability studies are presented in the online resource (Table S4).

Phosphorylated FLT3 decreased by approximately 40% compared with control phosphorylation levels in tumor samples within 1 h after single oral administration of 1–10 mg/kg gilteritinib (Fig. 2c), indicating target inhibition by gilteritinib. Although the decreased phosphorylation state observed in the 1 mg/kg treatment group had returned to the pre-treatment level by 24 h, the marked decrease in phospho-FLT3 seen with 10 mg/kg was maintained throughout the 24 h experimental period. Furthermore, the phosphorylation level of STAT5, a downstream target of FLT3, was almost completely abolished in tumors 4 h and 8 h after single administration of gilteritinib at doses of either 6 mg/kg or 10 mg/kg, respectively. STAT5 phosphorylation levels remained low 24 h post drug, approaching only 20% of baseline levels (Fig. 2d). A dose of 3 mg/kg gilteritinib also decreased phospho-STAT5 to <20% of baseline levels at 4 h and 8 h, but returned to approximately 50% by 24 h.

The effect on tumor burden of inhibiting FLT3 phosphorylation was assessed following 28 days of once-daily oral administration of gilteritinib. Significant growth inhibition of MV4–11 tumors was observed at 1 mg/kg/day (63% inhibition; *P* < 0.05) and 3 mg/kg/day (80% inhibition; *P* < 0.01), and near-complete tumor regression was seen at 6 mg/kg/day (93%; *P* < 0.001) and 10 mg/kg/day (100%; *P* < 0.001) (Fig. 2e). Four of the six mice in the 6 mg/kg/day group experienced complete tumor regression; all six mice in the 10 mg/kg/day group experienced complete tumor regression. Body weight was not affected by treatment with gilteritinib at any tested dose (Fig. 2f).

### Inhibitory activity of gilteritinib against FLT3 containing ITD ± D835Y or F691 L/I mutations

**Fig. 3:**
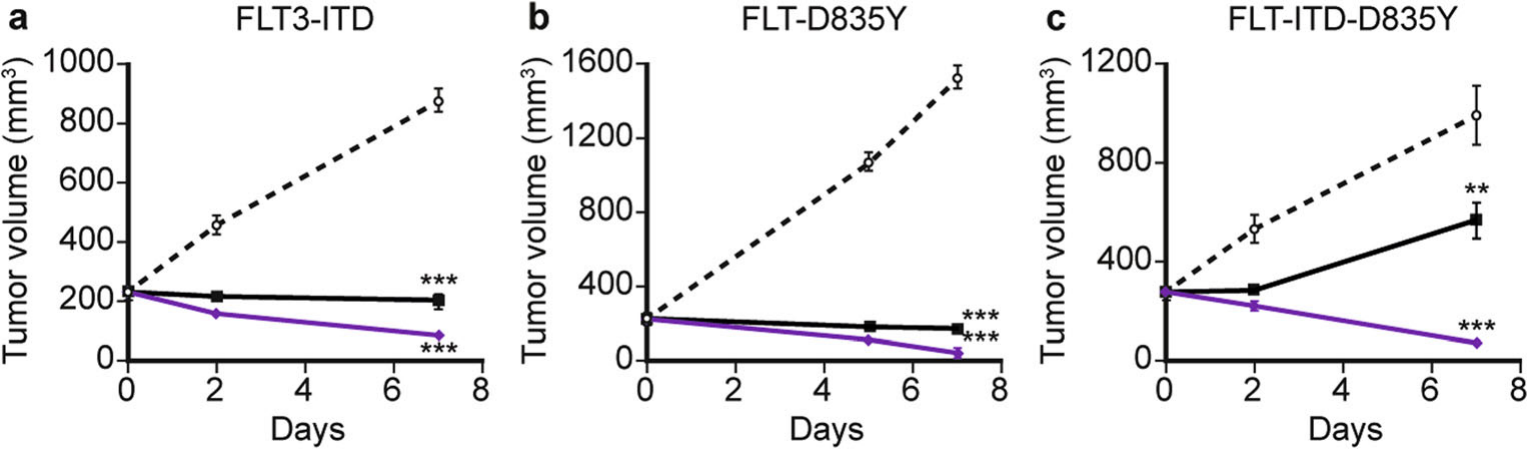
Gilteritinib induces regression of FLT3 mutant-expressing tumors in a mouse xenograft model. Male nude mice were xenografted with Ba/F3 cells expressing FLT3-ITD, FLT3-D835Y, or FLT3-ITD-D835Y. Following confirmed tumor growth, mice were treated with either 10 mg/kg or 30 mg/kg once-daily oral gilteritinib for up to 7 days. Tumor volume was assessed at specified time points in (**a**) FLT3-ITD xenografted mice, **b** FLT3-D835Y xenografted mice, and (**c**) FLT3-ITD-D835Y xenografted mice. Data presented are mean ± SEM for *n* = 5 mice/group. Symbols represent treatment groups: control (○), 10 mg/kg (■), and 30 mg/kg (). **P < 0.01 and ***P < 0.001 compared with the value of the control group on Day 7 (Dunnett’s test)

Since mutations within the TKD of FLT3 (eg, FLT3-D835Y or FLT3-F691) often confer resistance to FLT3 inhibitors that were previously effective against FLT3-ITD [21], the effect of gilteritinib on these resistance mutations was studied. Gilteritinib inhibited the cell growth of Ba/F3 cells expressing either FLT3-ITD, FLT3-D835Y, FLT3-ITD-D835Y, FLT3-ITD-F691 L, or FLT3-ITD-F691I, with IC_50_ values of 1.8 nM (95% CI: 1.0–3.0 nM), 1.6 nM (95% CI: 1.1–2.4 nM), 2.1 nM (95% CI: 1.4–3.0 nM), 22 nM (95% CI: 15–33 nM), and 49 nM (95% CI: 29–83 nM), respectively. The growth of parental Ba/F3 cells in the presence of IL-3 was inhibited with an IC_50_ value of 420 nM (95% CI: 350–500 nM), which represents the off-target inhibition of kinases other than FLT3. We confirmed that phosphorylation of FLT3 and its downstream targets STAT5, AKT, and ERK were inhibited by gilteritinib in a dose-dependent manner in Ba/F3 cells expressing either FLT3-ITD, FLT3-D835Y, or FLT3-ITD-D835Y (Fig. S2). Furthermore, in nude mice xenografted with Ba/F3 cells expressing either FLT3-ITD, FLT3-D835Y, or FLT3-ITD-D835Y, gilteritinib showed antitumor efficacy at 10 mg/kg and 30 mg/kg, and induced tumor regression at 30 mg/kg, in all three models (Fig. 3). Following treatment with quizartinib, a second-generation FLT3 inhibitor, the IC_50_ values for growth inhibition in Ba/F3 cells expressing FLT3-ITD, FLT3-D835Y, or FLT3-ITD-D835Y mutations were 0.46 nM (95% CI: 0.078–2.8 nM), 5.7 nM (95% CI: 1.5–21 nM), and 35 nM (95% CI: 9.3–130 nM), respectively.

### Gilteritinib binds to FLT3 at the ATP-binding site

**Fig. 4:**
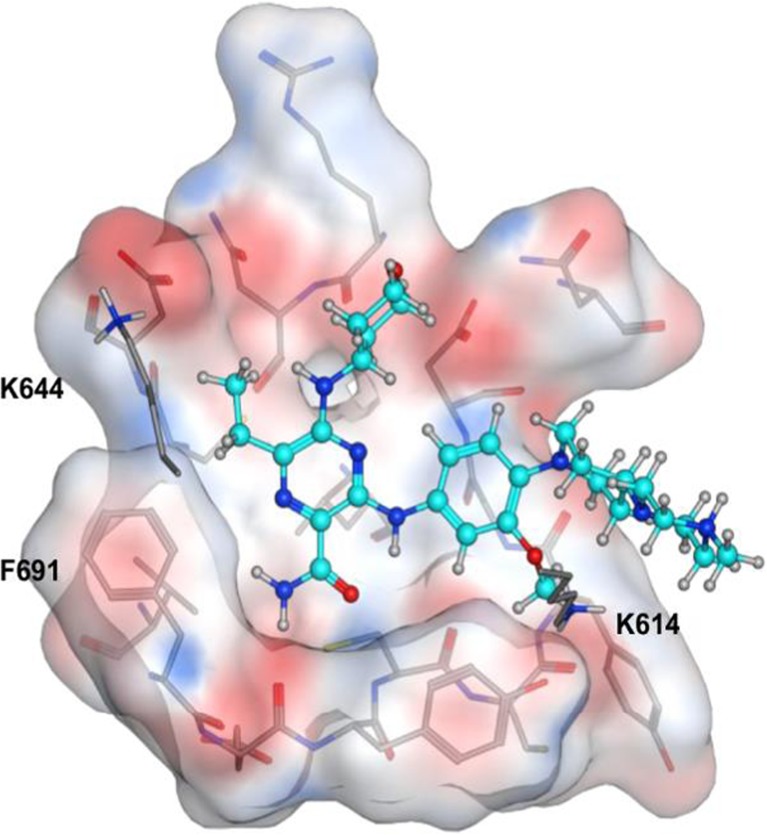
Computational modeling of gilteritinib binding to wild-type FLT3. Gilteritinib is shown as a ball-and-stick model. All of atoms are colored by the type of element (*white*: hydrogen; *cyan* and *gray*: carbon; *blue*: nitrogen; *red*: oxygen). The protein surface is colored by electrostatic potential (*blue*: positive; *red*: negative; *white*: neutral). For clarity, the hydrogen atoms of the protein are omitted with the exception of the polar hydrogen atoms of the side-chains of K614 and K644; similarly, the protein surface in front of the gatekeeper residue F691 is hidden

The finding that gilteritinib inhibited FLT3-D835Y and FLT3-ITD-D835Y, both of which harbor mutations in the activation loop essential for binding type 2 inhibitors, suggests that gilteritinib is a type 1 FLT3 inhibitor. To further understand the inhibitory activity of gilteritinib against mutated FLT3 at the structural level, computational modeling was performed for FLT3 with gilteritinib (Fig. 4). The modeling revealed that gilteritinib fits into the active (DFG [Asp-Phe-Gly]-in) conformation of FLT3 at the ATP-binding site, far from the D835 position in the activation loop. The modeling also showed that gilteritinib hydrophobically interacts with FLT3 at the F691 position.

### Gilteritinib prolongs survival in mice with IBMT of AML cells

**Fig. 5:**
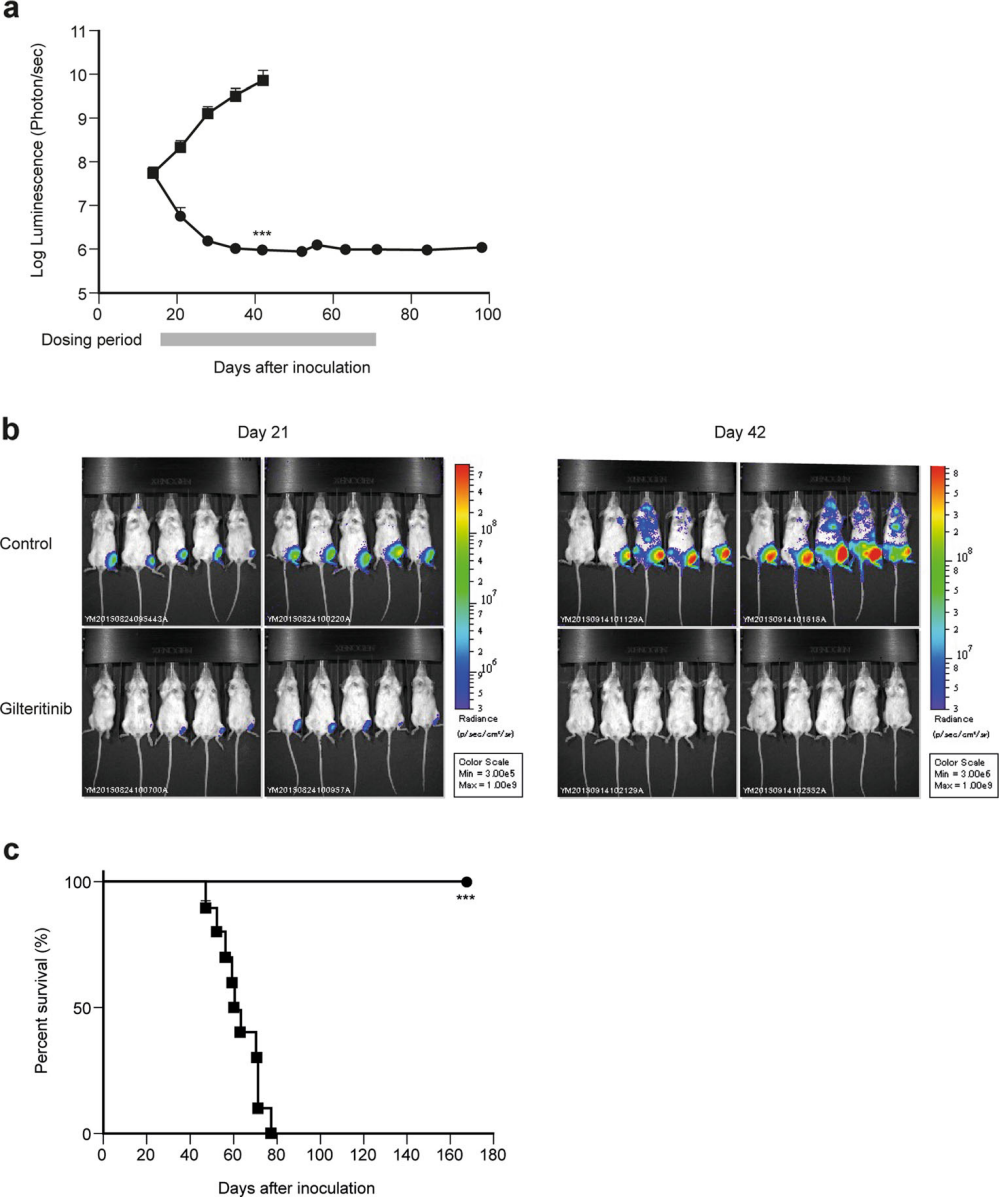
Gilteritinib significantly decreases leukemic burden and increases survival in an intra-bone marrow transplantation model of AML. Female NOD-SCID mice engrafted with MV4–11-luc cells were treated once daily with vehicle (■) or 30 mg/kg gilteritinib (●) for 56 days beginning on day 15. Bar represents treatment period. **a** MV4–11-luc cell bone marrow infiltration was monitored using whole-body imaging. Data presented as mean ± SEM for *n* = 10 mice/group. ***P < 0.001 compared with the value of the control group on day 42 (Student’s t-test). **b** Representative whole-body bioluminescence images from day 21 and day 42 are shown. **c** Kaplan–Meier analysis curve of mouse survival. ***P < 0.001 compared with the value of the control group (Log-rank test)

Treatment with gilteritinib showed a significant reduction in overall bioluminescence, with MV4–11-luc tumor growth dramatically reduced during the first 2 weeks of treatment (Fig. 5a), resulting in bioluminescence levels that were similar to that of background (around 10^6^ photons/s) on the penultimate day of treatment (day 70). Furthermore, when control mice showed MV4–11 cell infiltration in several areas of the body, gilteritinib-treated mice had no evidence of cell infiltration (Fig. 5b). Gilteritinib treatment significantly increased survival of MV4–11 xenografted mice and all treated mice survived to day 168, whereas vehicle control mice had a median survival time of 61.5 days (*P* < 0.001) (Fig. 5c).

## Discussion

These data demonstrate that gilteritinib, a FLT3/AXL TKI, shows potent efficacy in preclinical models of FLT3-mutated AML. In vitro kinase assays using gilteritinib revealed strong inhibition of FLT3 and AXL (in the nM range) and an 800-fold weaker inhibition of c-KIT than FLT3. Gilteritinib also inhibited phosphorylation of FLT3 with ITD and/or activation loop D835Y mutations in cellular assays. The inhibitory activity of gilteritinib against the D835Y mutation is further supported by computational modeling that showed gilteritinib binds to FLT3 within the activation loop far from the D835 site, allowing kinase inhibition to occur in the face of FLT3 mutations at this location. This potent FLT3 inhibition translated to a marked decrease in the cell viability of FLT3 mutation-positive cell lines with inhibition of several downstream targets. In vivo, gilteritinib was distributed at high levels in tumors after single oral administration and showed antitumor activity against FLT3-driven tumors in a mouse xenograft model. This antitumor activity was associated with a durable inhibition of phospho-FLT3 and phospho-STAT5, demonstrating the in vivo inhibitory effect of gilteritinib. Furthermore, treatment with gilteritinib decreased the leukemic burden and prolonged survival in a mouse IBMT model. Taken together, these data suggest that gilteritinib blocks FLT3 phosphorylation, impairs downstream signal transduction, and consequently inhibits AML cell proliferation in both in vitro and in vivo models.

These preclinical findings regarding the activity of gilteritinib suggest it may be a potential therapeutic treatment option for patients with FLT3-mutated AML. Although several TKIs have been investigated as potential therapies for AML, none have yet to be approved for clinical use [2, 9]. First-generation FLT3 inhibitors, such as sunitinib and sorafenib, are pan-kinase inhibitors that non-selectively target FLT3; this non-specificity often leads to off-target kinase inhibition and severe toxicity in patients [2]. Next-generation inhibitors, such as gilteritinib, crenolanib, and quizartinib, more selectively target FLT3, suggesting less off-target effects and potentially decreased toxicity in patients. The data presented herein suggest that gilteritinib has a potent inhibitory effect on FLT3 with minimal impact on c-KIT, demonstrating the specificity of the compound. Studies have shown that mice deficient in both *flt3* and c-*kit* show a large overall decrease in hematopoietic cell number, whereas mice deficient in only *flt3* have normal mature hematopoietic populations with specific deficiencies in primitive B lymphoid progenitors [22]. Thus, the targeted FLT3 inhibition and markedly weaker c-KIT inhibition by gilteritinib suggests a lower clinical risk of myelosuppression than often occurs with other TKIs [23].

When comparing the next-generation FLT3 inhibitors in development, all three compounds exhibit similar activity against FLT3-ITD in vitro, inhibiting the growth of MV4–11 cells at low nM concentrations (gilteritinib: 0.92 nM, crenolanib: 1.3 nM, quizartinib: 0.56 nM) [24, 25]. However, gilteritinib also showed potent inhibition of FLT3-D835Y and FLT3-ITD/D835Y mutations whereas quizartinib was ineffective at blocking these mutations [26]. Furthermore, we demonstrated that quizartinib had weaker activity against FLT3-D835Y and FLT3-ITD/D835Y mutations compared with FLT3-ITD mutations. Thus, in contrast to quizartinib, gilteritinib has the advantage of effectively blocking FLT3-ITD/D835Y and FLT3-D835Y. These preclinical data suggest that gilteritinib may provide improved clinical efficacy even in the presence of the D835Y, a mutation that has been clinically shown to confer resistance to FLT3 inhibitor treatment [27]. In addition, in cellular assays, gilteritinib showed inhibitory activity against F691 mutations, a mutation also detected in patients with AML who have relapsed following quizartinib treatment [26, 28]. However, in those assays, inhibition of cell viability for cells expressing FLT3-F691 was 10-fold to 20-fold weaker than for cells expressing FLT3-ITD, suggesting potential efficacy depending on the exposure level achieved in patients.

Although several similarities exist between gilteritinib and crenolanib (eg, effective inhibition of FLT3-D835Y and high selectivity for FLT3 compared with c-KIT), crenolanib treatment showed limited efficacy at the maximum tolerated dose in a xenograft mouse model in which MV4–11-luc cells were inoculated by intravenous injection, resulting in a significant survival benefit without a complete reduction in tumor cells [24]. By contrast, 30 mg/kg gilteritinib induced a significant reduction in bioluminescence from tumor cells to levels near background bioluminescence and improved survival such that all mice in the gilteritinib-treatment group remained alive during the experimental period. Furthermore, 30 mg/kg gilteritinib treatment continued to suppress the bioluminescence around 3 months post treatment, suggesting that this dose is also effective against minimum residual disease in bone marrow in this model. Overall, the cellular and animal model data suggest that gilteritinib may be advantageous compared with the other FLT3 inhibitors in development because it effectively blocks mutated FLT3 (both FLT3-ITD and FLT3-D835Y).

In addition to the strong preclinical inhibition of FLT3, gilteritinib also targets AXL. In vitro studies have shown that AXL is important for both wild-type and mutant FLT3 activation, suggesting that AXL may have a role in the pathobiology of AML [17]. Furthermore, preclinical evidence in FLT3-ITD expressing cells suggests that activation of AXL may be required for the development of acquired resistance to FLT3 inhibitors [19]. Additional in vivo studies have demonstrated that blocking AXL can suppress the growth of FLT3-ITD AML [17], decrease tumor size [16], and block the activation of cellular survival pathways while upregulating the apoptotic pathway [16]. Thus, the preliminary preclinical data presented herein, showing that gilteritinib inhibits AXL at concentrations similar to that for FLT3, suggest that dual inhibition of this pathway may lead to improved efficacy in the treatment of AML; however, it remains unclear whether AXL inhibition played a role in the tumor regression and improved survival induced by gilteritinib in the IBMT model.

Although these preclinical studies clearly demonstrate the potent activity of gilteritinib on FLT3 coupled with minimal activity against c-KIT, a few limitations should be noted. While these data are suggestive of a possible enhanced benefit to blocking both FLT3 and AXL, further studies are needed to fully characterize and understand the effect of gilteritinib on AXL in preclinical models thereby helping to fully elucidate the role of AXL in FLT3-mutated AML. In addition, understanding the effect of gilteritinib in cells from patients with AML may help shed light on the clinical implications of treatment with a FLT3-mutant inhibitor because the patient’s cells will be heterogeneous for the FLT3 mutation.

Overall, gilteritinib showed consistently potent inhibition of FLT3 in preclinical studies and demonstrated strong efficacy in FLT3-mutant tumor models. Furthermore, initial in vitro assays suggest that gilteritinib effectively blocks AXL activity while having minimal effect on c-KIT. Based on these data, it can be postulated that gilteritinib may lead to a reduced blast number and prolonged survival in patients with FLT3-ITD- or FLT3-D835Y-positive AML. This hypothesis requires confirmation in clinical studies.

## Electronic supplementary material

ESM 1 (DOCX 730 kb)
