# Supplementary material for: Gilteritinib, a FLT3/AXL inhibitor, shows antileukemic activity in mouse models of FLT3 mutated acute myeloid leukemia
Source: Invest New Drugs. 2017 May 17;35(5):556–65. doi: 10.1007/s10637-017-0470-z (PMC5613053; doi:10.1007/s10637-017-0470-z)
Supplement: Supplementary file 1 — (DOCX 730 kb) [file 10637_2017_470_MOESM1_ESM.docx]

# **Gilteritinib, a FLT3/AXL inhibitor, shows antileukemic activity in mouse models of FLT3 mutated acute myeloid leukemia**

**Authors:** Masamichi Mori^1^, Naoki Kaneko^1^, Yoko Ueno^1^, Masaki Yamada^2^, Ruriko Tanaka^1^, Rika Saito^1^, Itsuro Shimada^1^, Kenichi Mori^1^, Sadao Kuromitsu^1^

**Author Affiliations:** ^1^Drug Discovery Research, Astellas Pharma Inc., 21 Miyukigaoka, Tsukuba-shi, Ibaraki, Japan; ^2^Pharmacology Research Division, Astellas Research Technologies Co., Ltd, 21 Miyukigaoka, Tsukuba-shi, Ibaraki, Japan

**Corresponding Author:** Masamichi Mori, Research Program Management Office, Drug Discovery Research, Astellas Pharma Inc., 21 Miyukigaoka, Tsukuba-shi, Ibaraki 305-8585, Japan (Phone) +81-29-863-6584, (Fax) +81-29-852-2955, (E-mail) [masamichi.mori@astellas.com](mailto:masamichi.mori@astellas.com)

**SUPPLEMENTAL MATERIALS**

**Supplemental Methods**

**Cell viability assays**

To examine the effect of ASP2215 on cell viability, MV4-11, MOLM-13, and Ba/F3 cells expressing either FLT3-ITD, FLT3-D835Y, FLT3-ITD-D835Y, FLT3-ITD-F691L or FLT3-ITD-F691I were seeded into 96-well white plates (Nunc F96 MicroWell™ Plates, white; Thermo Fisher Scientific Inc., Waltham, MA) at 1000 cells/well in Iscove’s Modified Dulbecco’s Medium, 500 cells/well in RPMI 1640 or 1000 cells/well in RPMI 1640, respectively. All cell lines were cultured overnight in a humidified incubator at 37°C with 5% CO_2_ in media supplemented with 10% heat-inactivated FBS. Cell viability was determined for MV4-11 and MOLM-13 cells after 5 days of treatment with a dose course of gilteritinib; viability of Ba/F3 cells and cells expressing mutant FLT3 was assessed after treatment for 2 days with a dose course of gilteritinib and quizartinib. An additional study was conducted in Ba/F3 cells treated with both gilteritinib and IL-3 (1 ng/mL) to ascertain off-target effects of gilteritinib. Dose courses of gilteritinib for all cell viability assays are found in supplemental Table 4.

After treatment, the CellTiter-Glo^®^ Luminescent Cell Viability Assay (Promega) was used according to the manufacturer’s instructions and luminescence was measured using an ARVO-HTS plate reader (Perkin Elmer Inc., Waltham, MA). The assay was performed in either triplicate or quadruplicate. The effect of gilteritinib on cell proliferation was analyzed using SAS software (SAS Institute Inc., Cary, NC), Microsoft Excel (Microsoft), or GraphPad Prism (GraphPad Software), and the IC_50_ value of each experiment was calculated via Sigmoid-Emax model non-linear regression analysis. The geometric mean was calculated from three individual experiments.

**Preparation of Ba/F3 constructs**

The mutants of FLT3 (NM_004119), FLT3-D835Y (gat to tat), FLT3-ITD (insertion of acgttgatttcagagaatatgaatatgatc at 1799 position of CDS), FLT3-ITD-F691I (ttt to att), FLT3-ITD-F691L (ttt to ctt), FLT3-ITD-D835Y were encoded into pMX-puro vector (Cell Biolabs, Inc., San Diego, CA), and transfected with FuGENE-HD (Promega) into Platinum-E cells. The next day, the culture medium was changed and cultured for 72 h, and the culture medium were recovered and filtrated. Ba/F3 cells were infected with a retroviral vector for 6 h and cultured in the growth medium for 3 days. After the drug selection with puromycin at 1.5 μg/mL for 3 days, cells were cultured in the growth medium minus IL-3.

**FLT3 immunoprecipitation**

For the immunoprecipitation of FLT3, MV4-11 cells were seeded into 6-well plates at 5×10^6^ cells/well and cultured for 1 day prior to treatment. Following 2 h of treatment with either DMSO or a dose range of gilteritinib (0.1–10 nM), cells were lysed in RIPA buffer (Thermo Fisher Scientific) containing phosphatase / protease inhibitors, and the subsequent cell lysate was sonicated and centrifuged to pellet debris. Cell lysates (500 μg) were incubated with an anti-FLT3 antibody sc-480 (Santa Cruz Biotechnology, Santa Cruz, CA) overnight at 4°C and then incubated for 2 h with 20 μL Protein G Sepharose™ 4 Fast Flow (GE Healthcare, Fairfield, CT). The beads were washed with lysis buffer and boiled for 5 min in a 1 × SDS sample buffer (Wako Pure Chemical Industries, Osaka, Japan). Samples were electrophoresed on 7.5% gels and transferred to PVDF membranes.

**Detection of downstream FLT3 targets and AXL**

For the detection of ERK, STAT5, and AKT and their phosphorylated forms, MV4-11 cells were seeded at 1×10^6^ cells/well in 6-well plates (AGC Techno Glass, Shizuoka, Japan) and cultured for 1 day prior to treatment with either DMSO or a dose range of gilteritinib (0.1–10 nM). Cells were treated for 2 h prior to cell lysate preparation in RIPA buffer supplemented with phosphatase / protease inhibitors. Following sample preparation, protein lysates were electrophoresed on 7.5% gels and transferred to PVDF membranes.

For the detection of AXL and phospho-AXL, MV4-11-AXL cells were seeded at 1×10^6^ cells/dish in 10-cm dish and cultured for 1 day prior to treatment with either DMSO or gilteritinib doses ranging from 1 to 100 nM. Cells were treated for 4 h prior to cell lysate preparation in RIPA buffer supplemented with phosphatase / protease inhibitors. The protein lysates were subsequently electrophoresed on 7.5% gels and transferred to PVDF membranes.

**Immunoblotting**

All membranes were blocked using either Blocking One or Blocking One-P (Nacalai Tesque Inc., Kyoto, Japan) for 1 h prior to an overnight incubation with primary antibody. Membranes were incubated overnight at 4°C with one of the following antibodies: anti-FLT3 antibody (Abcam), anti-phosphorylated pan-tyrosine, anti-ERK, anti-phospho-ERK, anti-STAT5, anti-phospho-STAT5, anti-AKT, anti-phospho-AKT, anti-AXL, anti-phospho-AXL, or anti-actin. The membranes were then washed with 1× TBS Tween-20 (TBS-T) Buffer (Thermo Fisher Scientific Inc., Rockford, IL) and incubated with an appropriate HRP-linked secondary antibody (Cell Signaling Technology) for 1 h at room temperature. After a final wash, ECL Western Blotting Detection Reagent ECL-prime (GE Healthcare, Fairfield, CT) were applied to the membranes and signals were detected with a CCD camera (ImageQuant LAS4000; GE Healthcare). Signal intensity was then quantified using ImageQuant™ TL software (GE Healthcare). The study was performed once in triplicate, and the mean percentage of phosphorylated protein to total protein relative to that in vehicle-treated cells was calculated.

**In vivo mouse xenograft model**

For studies examining the in vivo kinase inhibitory activity of gilteritinib, male nude mice (CAnN.Cg-Foxn1nu/CrlCrlj[nu/nu]) (Charles River Laboratories Japan, Inc., Kanagawa, Japan), that were 5 weeks old, were subcutaneously inoculated with MV4-11 cells (5×10^6^ cells/mouse) on day 0. After 1 month, mice were divided into groups, with each group having approximately the same mean tumor volume. Mice with MV4-11 tumors were treated with a single oral dose of gilteritinib (1 mg/kg, 3 mg/kg, 6 mg/kg, or 10 mg/kg) and tumors were excised 1 h, 2 h, 4 h, 8 h, and 24 h after administration.

For in vivo xenograft studies on gilteritinib antitumor activity, MV4-11 cells (5×10^6^ cells/mouse) or Ba/F3 cells expressing a FLT3-ITD, FLT3-D835Y, or FLT3-ITD-D835Y mutation (3×10^6^ cells/mouse) were subcutaneously injected into the flank of male nude mice at 7 weeks old (MV4-11) or 4 weeks old (Ba/F3) (Charles River Laboratories). After tumor establishment, mice were divided into groups such that the mean tumor volume was approximately equal in each group. Mice were then orally administered either 0.5% methylcellulose or once-daily gilteritinib suspended in a 0.5% methylcellulose solution at doses of 1 mg/kg, 3 mg/kg, 6 mg/kg, and 10 mg/kg (MV4-11) or 10 mg/kg and 30 mg/kg (Ba/F3).

**ELISA for phosphorylated-FLT3 and total FLT3**

MULTI-ARRAY^®^ 96-well plates (Meso Scale Discovery, Rockville, MD) were coated with capture antibodies for phosphorylated FLT3 or total FLT3 (DuoSet^®^ IC, R&D systems, Minneapolis, MN) and incubated overnight at 4°C with shaking. After washing with TBS-T, coated plates were blocked with 3% MSD^®^ Blocker-A (Meso Scale Discovery) in TBS-T for 1 h at room temperature and then incubated with total protein lysates derived from MV4-11 xenografted tumors (500 μg/well for phosphorylated FLT3 and 20 μg/well for FLT3), overnight at 4°C. Following a wash step, anti-phosphotyrosine antibody (clone 4G10^®^, Biotin Conjugate [Millipore, Billerica, MA]) for the detection of phosphorylated FLT3 or the DuoSet^®^ IC FLT3 detection antibody for total FLT3 were added to the plate and incubated for 2 h at room temperature. Wells were then washed and incubated with MSD^®^ SULFO-TAG Labeled Streptavidin (Meso Scale Discovery) for 1 h at room temperature, followed by the addition of MSD^®^ Read Buffer (Meso Scale Discovery). Electrochemiluminescent signals were measured using a SECTOR^®^ imager (Meso Scale Discovery). Phosphorylation levels were normalized to total FLT3 levels in each group (3 mice/group except the point at 1 h after the administration of 1 mg/kg [2 mice]) relative to the mean value of the control group (5 mice/group). Data are reported as mean ± SEM.

**Immunoblot for phosphorylated-STAT5 and STAT5 in xenograft mouse model**

MV4-11 xenograft tumor protein samples were electrophoresed on 7.5% gels, transferred to PVDF membranes, and probed for phosphorylated-STAT and total STAT5 as described earlier with one alteration. Following detection of phosphorylated STAT5, the membrane was stripped using Restore™ Western Blot Stripping Buffer (Thermo Fisher Scientific) and then re-probed for total STAT5. Phosphorylation levels were normalized to total STAT5 levels in each group (3 mice/group) relative to the mean value of the control group (4 mice/group). Data are reported as mean ± SEM.

**Detection of phospho-FLT3 and downstream targets in Ba/F3 cells expressing mutant FLT3**

To detect phosphorylated FLT3 and its downstream targets in Ba/F3 cells expressing mutant FLT3, cells were seeded at 6×10^6^ cells per 10 cm dish and cultured overnight prior to treatment. Cells were treated with either DMSO or 0.1 nM, 1 nM, or 10 nM gilteritinib for 2 h. Cells were lysed in RIPA buffer and blotted for target proteins of interest as described earlier. Densitometry data normalized to total protein and then to the mean vehicle control for each target. Mean values reported from triplicate points.

**Supplemental Tables**

**Table S1** Inhibitory activity of gilteritinib against various tyrosine kinases

Inhibitory assay for the panel of tyrosine kinases was conducted using off-chip MSA. Percentage inhibitions were determined in a single experiment

**Table S2** Gilteritinib inhibition of phosphorylated STAT5, AKT, and ERK in MV4-11 cells

| Target | Kinase inhibition at the specified gilteritinib concentration (%) | | |
| --- | --- | --- | --- |
|  | 0.1 nM | 1 nM | 10 nM |
| p-STAT5 | 114 | 23 | 0 |
| p-AKT | 65 | 48 | 9 |
| p-ERK | 54 | 22 | 1 |

**Table S3** Pharmacokinetic parameters of gilteritinib after single oral dosing of gilteritinib in nude mice xenografted with MV4-11 cells

| Plasma concentration | | |
| --- | --- | --- |
| Dose (mg/kg) | C_max_ (ng/mL) | AUC_t_ (ng∙h/mL) |
| 1 | 6.558 | 25.20 |
| 6 | 45.90 | 269.0 |
| 10 | 83.01 | 492.8 |
| Intratumor concentration | | |
| Dose (mg/kg) | C_max_ (ng/g) | AUC_t_ (ng∙h/g) |
| 1 | 90.61 | 1186 |
| 6 | 772.1 | 12880 |
| 10 | 1125 | 17330 |

AUC_t_, area under the plasma concentration–time curve from time 0 h to the last determinable time point;
C_max_, maximum plasma concentration.

**Table S4** Gilteritinib dose ranges for cell viability studies

| Cell line | Gilteritinib dose range (nM) |
| --- | --- |
| MV4-11 cells | 0.03–30 |
| MOLM-13 cells | exp 1: 0.03–30  exp 2: 0.1–3000  exp 3: 0.03–300 |
| Ba/F3 wild-type cells | 180–3000 |
| FLT3-ITD_Ba/F3 | 0.05–12.8 |
| FLT3-D835Y_Ba/F3 | 0.05–12.8 |
| FLT3-ITD-D835Y_Ba/F3 | 0.05–12.8 |
| FLT3-F691L/I_Ba/F3 | 2.3–300 |

**Supplemental Figures**

**Supplemental Figure Legend**

**Fig. S1.** Gilteritinib inhibits phosphorylation of AXL in MV4-11-AXL cells. MV4-11-AXL cells exogenously expressing AXL were cultured overnight and treated with a dose course of gilteritinib for 4 h. Protein lysates were collected in RIPA buffer and immunoblotting was performed to detect both phosphorylated and total levels of AXL (triplicate). Densitometry was used to assess levels of phosphorylated AXL and total AXL protein to determine the percentage inhibition AXL phosphorylation with gilteritinib versus the control (vehicle) group.

**Fig. S2** Gilteritinib potently inhibits FLT3 and its downstream targets in Ba/F3 cells expressing mutant FLT3

Ba/F3 cells expressing mutant forms of FLT3 were cultured overnight and treated with a dose course of gilteritinib for 2 h. Protein lysates were collected in RIPA buffer and immunoblotting was performed to detect both the phosphorylated and total form of the target proteins FLT3, STAT5, AKT, and ERK. Immunoblot results from (a) Ba/F3 cells expressing FLT3-ITD, (b) Ba/F3 cells expressing FLT3-D835Y, (c) Ba/F3 cells expressing FLT3-ITD-D835Y. Densitometry was performed to determine the percentage inhibition of the phosphorylated form of each target in relation to the total protein compared with the vehicle control group. Data are presented as mean percentage inhibition; results are shown (triplicate)

**Fig. S1.** Gilteritinib inhibits phosphorylation of AXL in MV4-11-AXL cells

**Fig. S2** Gilteritinib potently inhibits FLT3 and its downstream targets in Ba/F3 cells expressing mutant FLT3

**
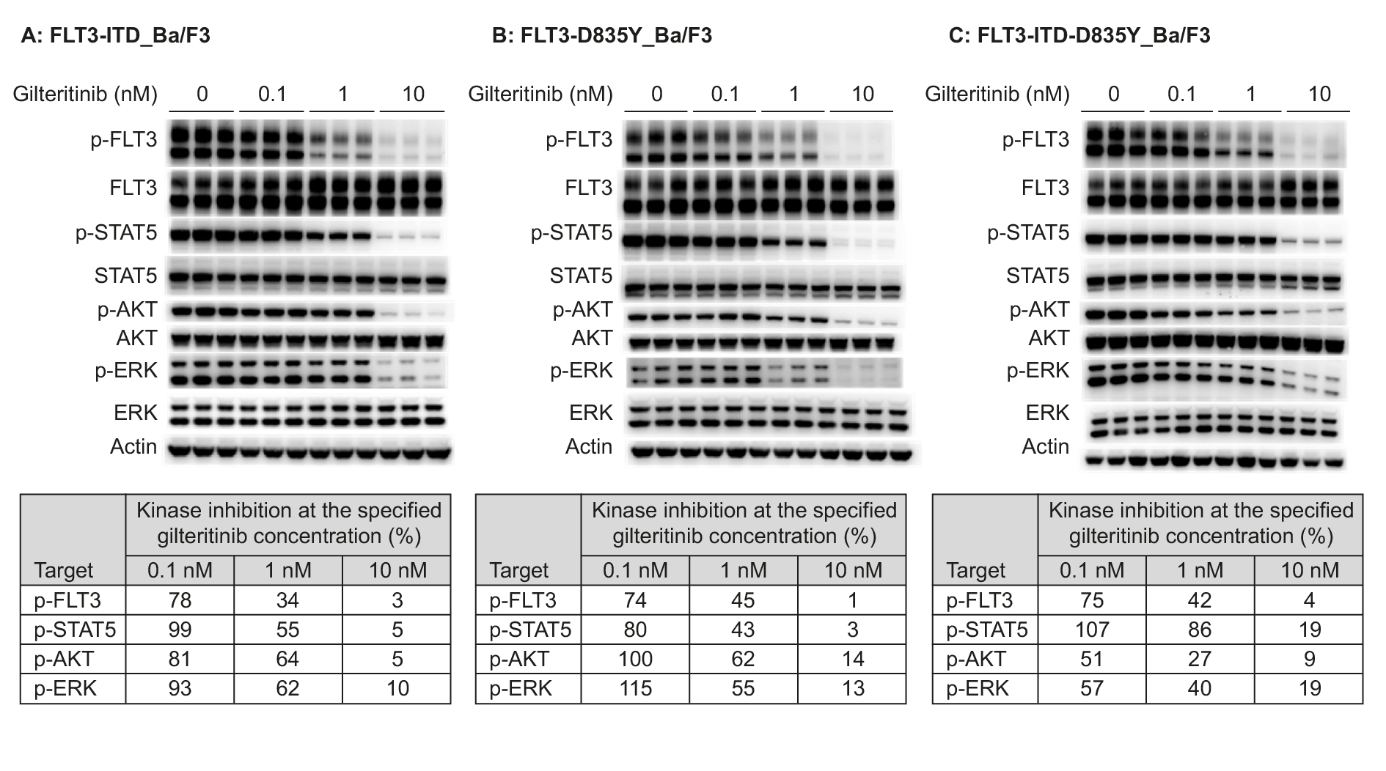
**
